# Expression, immunolocalization and serodiagnostic value of a myophilin-like protein from *Schistosoma japonicum*

**DOI:** 10.1016/j.exppara.2008.01.017

**Published:** 2008-05

**Authors:** Hailin Peng, Kai Song, Chengyu Huang, Sai Ye, Huaiguang Song, Wei Hu, Zeguang Han, Donald P. McManus, Guoping Zhao, Qinghua Zhang

**Affiliations:** a Shanghai Institute of Hematology, Ruijin Hospital, State Key Laboratory of Medical Genomics, Shanghai Jiao Tong University School of Medicine, Shanghai, China; b National Engineering Research Center of Biochip at Shanghai, Shanghai, China; c Chinese National Human Genome Center at Shanghai, Shanghai, China; d Institute of Parasitic Disease, Chinese Center for Disease Control and Prevention, Shanghai, China; e Department of Clinical Laboratory, Taizhou People’s Hospital, Jiangsu, China; f Molecular Parasitology Laboratory, Queensland Institute of Medical Research, Brisbane, Australia

**Keywords:** *Schistosoma japonicum*, Protein Sj myophilin-like, Myophilin-like protein, Schistosomiasis, Serodiagnosis, Immunofluorescence, ELISA, enzyme-linked immunosorbent assay, SDS–PAGE, sodium dodecyl sulfate–polyacrylamide gel electrophoresis, RT-PCR, reverse transcription-polymerase chain reaction, OCT, optimal cutting temperature, PVDF, polyvinylidene difluoride, PBST, phosphate buffer solution Tween, AP, alkaline phosphatase, HRP, horseradish peroxidase, IPTG, isopropyl-β-d-galactopyranoside, FITC, fluorescein sothiocynate

## Abstract

The cDNA of a *Schistosoma japonicum* myophilin-like protein was cloned, sequenced, and expressed in *Escherichia coli* as a recombined protein (rSj myophilin-like protein), and the protein was purified by affinity chromatography. The deduced amino acid sequences of the Sj myophilin-like protein showed significant homology to myophilin, calponin, Np22 and Mp20. Northern blot and RT-PCR analyzes revealed expression of the Sj myophilin-like protein mRNA in eggs, sporocysts, cercariae, hepatic schistosomula and adult worms. Confocal fluorescence microscopy localized the native protein to the muscle of the adult worm. In schistosome-infected rabbits, the rSj myophilin-like protein antibody level, assessed by ELISA, was elevated after infection but was reduced after praziquantel treatment. In humans, the myophilin-like protein antibody level was evaluated by ELISA in sera from 33 non-infected humans and 61 schistosomiasis patients; the results showed a highly significant difference between the two groups with a sensitivity of 57.4%. Taken together, the myophilin-like protein may prove useful for monitoring the therapeutic effect of praziquantel rather than in serodiagnosis of schistosomiasis.

## 1 Introduction

Schistosomiasis remains a serious public health problem worldwide, infecting more than 200 million people, mostly in tropical regions, and is endemic in 74 developing nations. *Schistosoma japonicum* is endemic in China and the Philippines, and is also found in Sulawesi, Indonesia (Savioli et al., 2002; Ross et al., 2002; Engels et al., 2002; Chitsulo et al., 2004). Correct diagnosis is fundamental for controlling schistosomiasis, including case detection and resulting community treatment, assessment of morbidity and the evaluation of control strategies. At present, parasitological and immunological tests are considered as the main methods in schistosomiasis diagnosis. The appearance of parasite eggs in urine or feces directly indicates the presence of the worms. However, disadvantages of this relatively time-consuming approach are well recognized, including high fluctuation in egg counts and easily missed low infections, (Kongs et al., 2001; Ross et al., 2001). Immunodiagnostic techniques, on the other hand, have the advantages of much higher sensitivity and ease of performance (Ross et al., 2001; Bergquist, 1992; Wu, 2002) and many antibody assays have been developed (Xiao et al., 2003; van Dam et al., 2004; Stothard et al., 2006). However, few serological tests are commercially available, and the preparation of antigens from parasites requires the maintenance of the complete parasitic cycle and often tedious laboratory extraction procedures. In the past decade, recombinant schistosome antigen-based serodiagnostic assays have been developed (Moser et al., 1992; Liu et al., 1993; Hu et al., 2003a,b), but more specific and sensitive antigen preparations are required to develop assays with improved performance. In addition, although praziquantel-based chemotherapy (Gönnert and Andrews, 1977; Kihara et al., 2007) is the best regimen available today for the clinical management of schistosomiasis and is highly effective against all of the human schistosomes (Geerts and Gryseels, 2000; Kihara et al., 2007), few simple and effective methods for monitoring the therapeutic effect of praziquantel are available.

Using data from the published genomic and cDNA sequences for *S. japonicum* (Hu et al., 2003a,b), we identified a gene encoding a myophilin-like protein (GenBank Accession No.: AY223463). Here we report the prokaryotic expression and purification of this *S. japonicum* myophilin (rSj myophilin), its functional evaluation of its potential rabbit models. In addition, immunolocalization of the Sj myophilin-like was reported.

## 2 Materials and methods

### 2.1 Preparation of schistosome samples for analysis

A Chinese isolate of *S. japonicum* was collected from Anhui province, China, and the life cycle was maintained in *Oncomelania hupensis* snails and Kunming strain mice at the Institute of Parasitic Disease, Shanghai. mRNA samples from eggs, sporocysts, cercaria, hepatic schistosomula and adult worms, and the native soluble proteins from adult worms of *S. japonicum* were prepared as previously described (Hu et al., 2003a,b; Liu et al., 2006, 2007).

### 2.2 Sequence similarity comparison

Online BLAST was used to find homologous sequences of the *S. japonicum* myophilin-like cDNA and protein. The sequences were aligned using CLUSTAL(1.0) multiple sequence alignment programs (http://us.expasy.org/).

### 2.3 Construction of recombined plasmid pET22b-Sj myophilin-like and pBluescript-Sj myophilin-like

The *S. japonicum* myophilin-like cDNA was PCR amplified with primers flanked with BamHI and XhoI (F 5′-GCGTCAG*GGATCC*GATGGCTAATCAACCT TTAG-3′, R 5′-GCGTCTG*CTCGAG*ATCAAGAATCATACGTTG-3′) from the *S. japonicum* cDNA clone pBluescript II SK(+) sjs2-011 C10 and subcloned into the pET22b expression vector and pBluescript II SK(+), respectively. The subcloned protein coding sequences (CDS) were verified to be mutation free by sequencing.

### 2.4 Heterogeneous expression and purification of rSj myophilin-like protein

Recombinant CDS expression was performed following published procedures (Sambrook and Russell, 2001) with minor modifications. Briefly, the recombinant plasmid pET22b-Sj myophilin-like was transformed into *Escherichia coli* BL21 (DE3), and then the cells were grown, reaching an optical density of 0.6 at 600 nm, and induced with 0.8 mM IPTG at 30 °C for an additional 4 h. After incubation, the culture was harvested and processed according to published protocols (Marshak et al., 1996) to obtain a pellet of inclusion bodies. Then the inclusion bodies were dissolved in loading buffer containing 6 M guanidine hydrochloride, after centrifugation at 4 °C (8000 rpm, 30 min), the supernatant was filtered through a 0.45-μm-pore-size filter before being loaded onto a Chelating Sepharose Fast Flow column (GE Healthcare, USA). After washing the column, the adsorbed recombinant protein was eluted using four steps of increased imidazole concentration in elution buffer. Fractions were analyzed by SDS–PAGE and stained with Coomassie blue to assess their purity.

### 2.5 RT-PCR

Total RNA (1–5 μg) from each *S. japonicum* stage was reverse-transcribed using a SuperScript™ II Reverse Trancriptase system (Invitrogen, USA) and oligo (dT). The resulting cDNAs and specific oligonucleotide primers (F 5′-GCGTCAG*GGATCC*GATGGCTAATCAACCTTTAG-3′ and R 5′-GCGTCTG*CTCGAG*ATCAAGAATCATACGTTG-3′) were used in a PCR reaction to amplify a 570 bp fragment using an EX-Taq DNA polymerase system (TAKARA, Dalian, China). The PCR products were analyzed on a 1% agarose gel.

### 2.6 Northern blot analysis

Northern blot analysis was performed using a non-Isotopic DIG Northern Starter Kit (Roche Diagnostics, USA) as previously described (Li et al., 2006). Specifically, 3 μg of total RNA (from adult male worms) per lane were loaded for electrophoresis and the pBluescript-Sj myophilin-like plasmid was linearized with XbaI for probe generation.

### 2.7 Preparation of polyclonal mouse anti-Sj myophilin-like serum

Polyclonal mouse antisera were prepared by genetic-immunization using a DNA prime-protein boost strategy (Peng et al., 2007). Briefly, mice (Balb/c, female, 6 weeks) were injected intramuscularly 3 times with 100 μg recombined-plasmid pVAX1-Sj myophilin-like followed by boosting with 50 μg of rSj myophilin-like protein at 2 weeks intervals. Serum was collected 3 days after the final boost.

### 2.8 Immunofluorescence assay

Immunofluorescence assays were performed as previously described (Liu et al., 2006) with some modifications. Four-micron-thick frozen sections of adult worms were embedded in OCT compound and fixed with methanol for 20 min at room temperature. Slides were blocked with goat serum overnight at 4 °C, and incubated in a humid atmosphere at room temperature for 2 h with the anti-Sj myophilin-like serum (1:400 in 0.1% PBST) or pre-immunized mouse serum. The slides were washed twice and incubated for 2 h with 1:80 FITC-conjugated goat anti-mouse IgG (Sigma, USA), containing 20 μg/ml Evans blue in a humid atmosphere2 at room temperature. Confocal fluorescence microscopy (Zeiss 510 Meta, German) was used in visualization of antibody staining.

### 2.9 Human and rabbit sera

Human sera were obtained from 61 patients with schistosomiasis japonica, confirmed by Kato–Katz stool examination (Katz et al., 1970), from endemic area in Jiangxi Province. Control sera were obtained from 33 volunteers without schistosomiasis from a non-endemic area in Shanghai.

Seventeen rabbits were exposed to an average of 1000 cercariae per rabbit; nine of these infected rabbits were treated with praziquantel (administered orally at a dose of 200 mg/kg body weight) 10 weeks after infection. Serum samples from the rabbits were collected by lateral ear vein puncture at 3 weekly intervals.

### 2.10 Western blotting

SDS–PAGE (10%) was used to analyze the recombinant or native Sj myophilin-like proteins. Aliquots of purified rSj myophilin-like (50 ng/well) or crude extracts (10 μg/well) were loaded onto gels. After electrophoresis, the gels were either Coomassie blue-stained to visualize the protein bands or electro-transferred onto PVDF membranes (Millipore, USA) and reacted with antibody probes. PVDF membranes were blocked for 1 h in 0.1%-PBST containing 5% milk powder and immunostained with human sera (1:100 in 0.1%-PBST) or mouse polyclonal antiserum (1:4000 in 0.1%-PBST). Then, the membranes were washed 3 times in 0.1%-PBST for 15 min and incubated with affinity purified HRP-conjugated goat anti-human or anti-mouse IgG (1:5000, Sigma, USA) and developed in an ECL Western blotting system (GE Healthcare, USA).

### 2.11 ELISA

The human and rabbit sera were also tested for specific IgG antibody levels by ELISA using plates coated (1 μg/ml) with the rSj myophilin-like purified protein. The secondary antibody used in the ELISA was AP-conjugated goat anti-mouse or goat anti-human IgG (Sigma, USA). Color reaction was developed by the addition of *o*-phenylenediamine (OPD) (Sigma, USA) in phosphate buffer and stopped with sulfuric acid. The absorbance was read at 490 nm using a micro plate reader.

The ELISA data were statistically analyzed by a group *t*-test, and a *P*-value <0.05 was regarded as significant.

## 3 Results

### 3.1 Molecular characterization of Sj myophilin-like protein

**Fig. 1:**
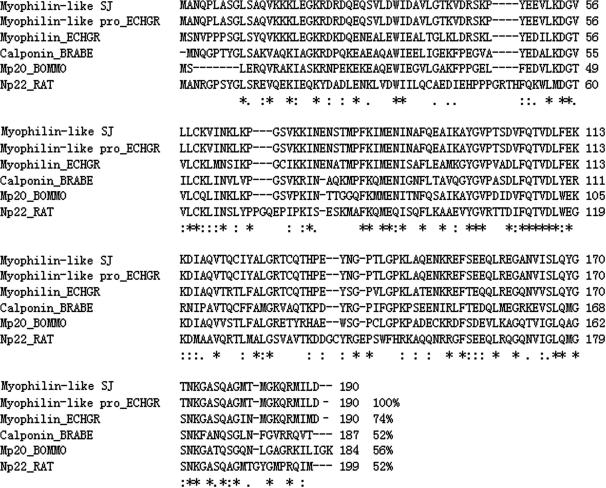
Multiple alignment of the deduced amino acid sequences of the Sj myophilin-like protein with the homologs, *E. granulosus* myophilin-like protein, myophilin antigen from *E. granulosus*, calponin of *B. belcheri*, Mp20 from *B. mori*, and neuronal protein 22 from *R. norvegicus.* Gaps are represented by dashed lines. “*” means that the residues in that column are identical in all sequences in the alignment; “:” means that conserved substitutions have been observed; “.” means that semi-conserved substitutions are observed.

The CDS encoding the Sj myophilin-like protein was successfully subcloned from the cDNA clone pBluescript II SK(+) sjs2-011 C10 into pET22b. The Sj myophilin-like clone was 570 bp in length, encoding a protein of 190 amino acid residues (Fig. 1), with a molecular mass of 21.138 kDa, and its isoelectric point (PI) was 8.66 (http://us.expasy.org/cgi-bin/pi_tool). The amino acid sequence of Sj myophilin-like is 100% identical to that of *Echinococcus granulosus* myophilin-like protein (GenBank AAT46028), and 74% identical to the myophilin antigen from *E. granulosus* (GenBank CAA82316), 52% identical to the calponin of *Branchiostoma belcheri* (GenBank BAC16745), 52% identical to the neuronal protein 22 from *Rattus norvegicus* (GenBank AAL66341), and 56% identical to the mp20 from *Bombyx mori* (GenBank NP_001040476) (Fig. 1).

### 3.2 Transcript analysis of Sj myophilin-like

**Fig. 2:**
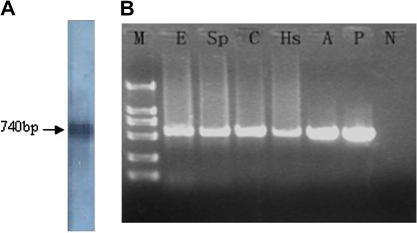
Gene expression analysis of Sj myophilin-like transcripts. (A) Northern blot analysis of Sj myophilin-like mRNA expression in *S. japonicum* adult worms. Northern blot with 3 μg total RNA from adult male worms. The transcript of the Sj myophilin-like protein band at 740 bp. (B) RT-PCR analysis of Sj myophilin-like mRNA in different developmental stages. M, molecular weight marker (2000, 1000, 750, 500, 250, 100 bp); E, eggs; Sp, sporocysts; C, cercariae; Hs, hepatic schistosomula; A, adults (mixed-gender); P, positive control, plasmid pET22b-Sj myophilin-like as template; N, no template control. cDNA samples used were from individual stage cDNA libraries.

To identify the transcript of the myophilin-like protein in adult worms, a Northern blot was performed with total RNA from adult male worms. A distinct signal of approximately 740 bp was observed (Fig. 2A). The size of the signal corresponds with the molecular size of the full-length cDNA clone found in the library.

RT-PCR was performed with various stages in order to detect the expression of myophilin-like mRNA during the *S. japonicum* life cycle. As shown in Fig. 2B, the expected size (∼570 bp) myophilin-like fragments were amplified from all of the cDNA libraries. The RT-PCR results showed that the myophilin-like mRNA was expressed in eggs, sporocysts, cercariae, hepatic schistosomula and adult worms.

### 3.3 Expression and purification of rSj myophilin-like protein

**Fig. 3:**
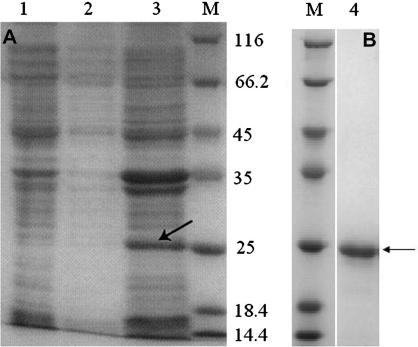
Expression analysis of the recombinant Sj myophilin-like protein. Lane M, molecular mass standards. (A) Lane 1, before induction; lane 2, supernatant after induction; lane 3, precipitate after induction. (B) Lane 4, purified protein after affinity chromatography.

IPTG (0.8 mM) efficiently induced expression of the rSj myophilin-like protein and the protein accumulated in inclusion bodies (Fig. 3A). The apparent molecular mass of the rSj myophilin-like protein was about 25 kDa. This band is about 3 kDa bigger than the estimated size deduced from the sequence data because the recombinant protein contains an additional 30 amino acids comprising the N-terminal pelB leader and the C-terminal 6× His tag sequence. The recombinant protein was subsequently purified using an affinity chromatography purification kit. It was absorbed by the Ni^2+^ through its 6× His tag when passed through the column. The absorbed proteins were eluted with a linear imidazole gradient as described in Section 2 (Fig. 3B, lane 4).

### 3.4 Western blots of Sj myophilin-like protein probed with human or mouse sera

**Fig. 4:**
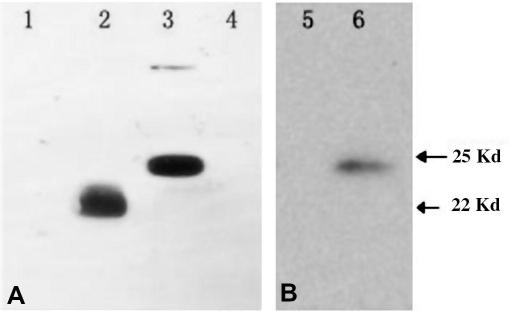
Western blot analysis of Sj myophilin-like protein. The samples loaded were crude protein extract from adult worms (lanes 1 and 2) or rSj myophilin-like protein (lanes 3–6). (A) Probed with mouse sera. Lanes 1 and 4 probed with pre-immune mouse serum; lanes 2 and 3 probed with sera from immunized mice. (B) Probed with human sera. Lane 5 probed with serum pool from healthy humans; lane 6, probed with serum pool from schistosomiasis japonica patients. An arrow indicates the location of the rSj myophilin-like protein (25 kDa) or native Sj myophilin-like protein (22 kDa).

Purified rSj myophilin-like protein (50 ng/well) and crude extract protein from adult worm (10 μg/well) were loaded onto SDS–PAGE gels. After electrophoresis and transfer, the proteins were probed with human or mouse sera. (Fig. 4). Pooled sera from healthy humans did not recognize the rSj myophilin-like protein (Fig. 4B, lane 5). In contrast, the rSj myophilin-like protein was recognized by a serum pool from schistosomiasis patients (Fig. 4B, lane 6). Polyclonal mouse antisera recognized a band of ∼22 kDa in the crude protein extract from adult worms (Fig. 4A lane 2), and this antiserum also recognized the rSj myophilin-like protein as a band about 25 kDa (Fig. 4A, lane 3). Neither the crude protein extract nor the recombinant protein reacted with the pre-immune mouse serum (Fig. 4A, lanes 1 and 4).

### 3.5 Immunofluorescence localization of Sj myophilin-like protein in adult worms

**Fig. 5:**
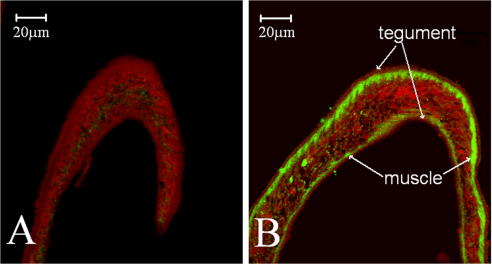
Immunolocalization of the Sj myophilin-like in adult male *S. japonicum*. (A) Detected with pre-immunized mouse sera; (B) Detected with mouse anti-serum against Sj myophilin-like protein. Magnification 400×.

The distribution of rSj myophilin-like protein in adult worms was investigated by indirect immunofluorescence. Cryosectioned *S. japonicum* adults were incubated with mouse anti-serum and pre-immune mouse serum as control. The myophilin-like protein was found to be distributed in the muscle of adult worms (Fig. 5). No fluorescence was detected in control sections.

### 3.6 Sj myophilin-like protein antibodies in human sera

A series of ELISA experiments were conducted to detect antibody levels in human sera using the rSj myophilin-like protein as antigen (Table 1). Two of the 33 healthy human serum samples were positive, giving a false positive rate of 6.06% (2/33). Sera (35 of 61) from confirmed schistosomiasis patients, were positive, giving a sensitivity of 57.4% (35/61). The difference between the two groups was statistically significant (*P* < 0.0001).

### 3.7 Diminution of antibody levels in treated infected rabbits

**Fig. 6:**
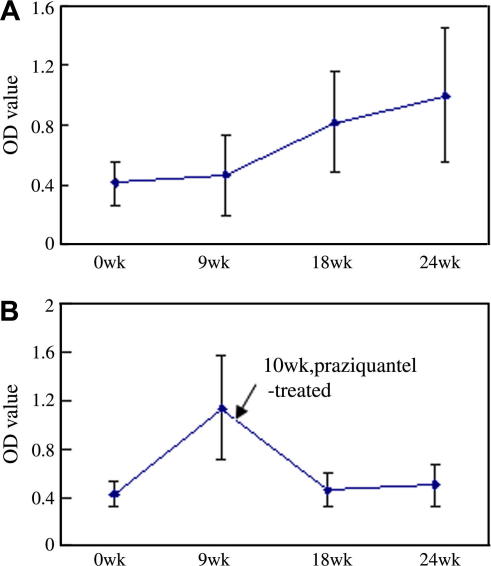
Changes in anti-Sj myophilin-like serum antibody levels in infected rabbits groups. (A) Group without treatment with praziquantel post-infection; (B) Group treated with praziquantel at 10 weeks post-infection.

Specific antibody levels in infected rabbits were determined over time by ELISA using the rSj myophilin-like protein as antigen (Fig. 5). In the praziquantel-treated group, the antibody level increased remarkably 9 weeks after treatment but then decreased to a level almost equal to that of the pre-infected animals (*P* > 0.05, data not shown) 11 weeks after treatment (Fig. 6B). In contrast, the antibody level of the un-treated group increased throughout the experiment until 18 weeks after infection, and the level was clearly different from that of the pre-infected animals (*P* < 0.05) (Fig. 6A).

## 4 Discussion

Myophilin was first identified as a muscle-specific protein of *E. granulosus*, where it appeared to be involved in the regulation of muscle contraction (Martin et al., 1995, 1996). A myophilin-like protein was subsequently identified from *E. granulosus* with high similarity (74% amino acid sequence identity) myophilin. In the present study, a myophilin-like protein from *S. japonicum* was identical to the ortholog from *E. granulosus.* By mapping the draft *S. japonicum* genome sequence, single copy of the myophilin-like protein gene was annotated in the *S. japonicum* genome; it contains four exons segregated by three introns (data not shown). No homolog of the *E. granulosus* myophilin antigen was identified within the *S. japonicum* genome (http://lifecenter.sgst.cn), a situation similar to *Schistosoma mansoni* (Haas et al., 2007; Oliveira, 2007).

To investigate transcription of the *S. japonicum* myophilin-like protein in different life cycle stages, Northern blotting and RT-PCR analysis were undertaken and the results revealed that transcripts of the myophilin-like protein were present in eggs, sporocysts, cercariae, hepatic schistosomula and adult worms. This corroborated earlier proteomics analysis which showed that the myophilin-like protein is present in the eggs, cercariae, schistosomula and adult worms of *S. japonicum* (Liu et al., 2006). We detected the myophilin-like protein in the muscle of adult worms by immunofluorescence. Taken together, it appears that the *S. japonicum* myophilin-like protein is a muscle protein and likely plays an important role throughout the life cycle in the developmental biology of the worm, similar to that reported for its homolog, calponin, in *B. belcheri* (Urano et al., 2003).

The antigenity of rSj myophilin-like protein was examined by Western blotting, and the level of anti-Sj myophilin-like antibody in sera was assessed by ELISA in uninfected individuals, schistosomiasis patients, infected rabbits, including animals treated with praziquantel. The ELISA results showed that the difference in anti-myophilin-like protein antibodies between healthy and schistosomiasis patients was significant (*P* < 0.0001), and a diagnostic sensitivity of 57.4% (35/61) was obtained. Although, for diagnosis, the sensitivity of the rSj myophilin-like protein is insufficiently high, it could be useful when combined with other antigen(s). In rabbits, the anti Sj myophilin-like protein antibody levels significantly increased after *S*. *japonicum* infection. In the group treated with praziquantel, the antibody level had decreased 10 weeks after infection, to the pre-infection level, whereas it increased continually in un-treated groups. Together with the antibody level differences between healthy and schistosomiasis patients, these results suggest that detection of anti-*S. japonicum* myophilin-like antibodies could be used to monitor the treatment efficacy of praziquantel.

In conclusion, we identified a novel muscle myophilin-like protein from *S. japonicum*. The rSj myophilin-like protein may be useful as an adjunct immunodiagnosis antigen for schistosomiasis as well as a marker of drug treatment efficacy.

## Figures and Tables

**Table 1:** Anti-Sj22 antibody detection in sera from healths human and schistosomiasis patients

Groups	OD_490_ value $\left(\overset{¯}{x}\pm \text{SD}\right)$	Positive cut-off$\left(\overset{¯}{x}+2\text{SD}\right)$	No. samples	Positives no. **(**%)	*P*-value⁎
Healths	0.2085 ± 0.0598	0.328	33	2 (6.06)	<0.0001
Patients	0.4312 ± 0.2291		61	35 (57.4)	

⁎ Group *t*-test.
